# Fuzzy Filtering Method for Color Videos Corrupted by Additive Noise

**DOI:** 10.1155/2014/758107

**Published:** 2014-02-06

**Authors:** Volodymyr I. Ponomaryov, Hector Montenegro-Monroy, Luis Nino-de-Rivera, Heydy Castillejos

**Affiliations:** Instituto Politecnico Nacional, ESIME (Culhuacan), Avenida Santa Ana 1000, Colonia San Francisco Culhuacan, 04430 Ciudad de Mexico, DF, Mexico

## Abstract

A novel method for the denoising of color videos corrupted by additive noise is presented in this paper. The proposed technique consists of three principal filtering steps: spatial, spatiotemporal, and spatial postprocessing. In contrast to other state-of-the-art algorithms, during the first spatial step, the eight gradient values in different directions for pixels located in the vicinity of a central pixel as well as the R, G, and B channel correlation between the analogous pixels in different color bands are taken into account. These gradient values give the information about the level of contamination then the designed fuzzy rules are used to preserve the image features (textures, edges, sharpness, chromatic properties, etc.). In the second step, two neighboring video frames are processed together. Possible local motions between neighboring frames are estimated using block matching procedure in eight directions to perform interframe filtering. In the final step, the edges and smoothed regions in a current frame are distinguished for final postprocessing filtering. Numerous simulation results confirm that this novel 3D fuzzy method performs better than other state-of-the-art techniques in terms of objective criteria (PSNR, MAE, NCD, and SSIM) as well as subjective perception via the human vision system in the different color videos.

## 1. Introduction

Image filtering has a wide field of applications such as image processing, computer vision, telecommunications, medicine, satellite imaging, and robots, where the main objective of the denoising procedure is to detect, filter, or remove undesired noise from a color image and videos. There exist different reasons why such noise arises, such as nonuniform lighting, random fluctuations in an object's surface orientation and texture, sensor limitations, and nonideal transmission. Noise affects not only the performance of an image in a specific task but also its perceived quality [1–3]. The most common type of noise encountered in practice is the additive noise that is generally assumed to be a stochastic process with a zero-mean Gaussian distribution and known variance *σ* ^2^ and in most cases spatially independent. The additive model is most appropriate when the noise in the model is independent of an image. The principal difference in contamination by additive noise is that every pixel of an image is corrupted; nevertheless, such pixels can be recovered by subtracting the random additive error. There exist other kinds of noise, such as speckle noise common in ultrasonic and SAR imaging and impulsive noise [4, 5]. There are many techniques for filtering different types of noise, among which the most important is to design an adaptive algorithm that considers the local information of texture, edges, and color features of an image. Some of the techniques commonly used in the filtering practice are based on the concept of adaptive vector median filtering [6–8]. Efficient filtering should be performed by obtaining a valid weighting procedure for the pixels in the vicinity of a central one that should be denoised [9–17].

An important difference between image and color videos filtering is that in videos it is possible to use previous (and/or future) frames for better pixel denoising in the actual frame. However, when two or more frames are processed together for noise removal local motions should be compensated between different frames, because in an other case they can introduce motion blur and ghosting artifacts. The literature reveals many approaches focused on this task, which is performed to match a central pixel with neighboring ones in a sliding window of the current frame with the most similar central pixel and neighboring pixels in an analogous window located in the neighborhood of the neighboring frames [18–24]. Additionally, there exists a high level of correlation between the neighboring frames in a video when local temporal motions can be considered to be very small. This gives an excellent opportunity to increase the number of pixels with high similarity in the spatiotemporal stage of filtering, increasing denoising quality for videos [25, 26]. Other filtering algorithms are focused on wavelet-based video encoders and filters that estimate motions, obtaining better performance than the spatial domain denoising techniques [24, 27–35].

Fuzzy-based filters for the reduction of additive noise and other kinds of noises (mainly impulsive noise) in color videos have been successfully applied as well [27, 36, 37]. The advantage of fuzzy filtering techniques is in the efficient preservation of image features, such as edges, chromaticity characteristics, texture, and fine details, while corrupted pixels are being filtered. Fuzzy logic filters are established by *membership functions* and *fuzzy rules* based on human knowledge and the ability to adapt their characteristics to the current image and noise [38–46].

In the literature, there are reports of denoising techniques that employ only temporal [47] or spatiotemporal information [36, 43, 48].

Several promising filtering procedures have been developed over the past five to six years, demonstrating good performance quality. These algorithms have shown sufficiently good performance results in removing additive noise, exhibiting good preservation of edges, textures, sharpness, and the chromatic properties of the filtered color image or videos. Let us present a short review of some of these techniques. In a later section, the best of these techniques are compared with the novel fuzzy filtering approach.

Yin et al. [26] report a linear minimum mean squared error (LLMMSE) spatiotemporal filter with adaptive motion compensation. This technique increases the performance quality of the 3D-LLMMSE filter. The spatiotemporal adjacent homogeneous pixels that match correctly the current pixel are included in the filtering data for the noise reduction; on the other hand, the outlier pixels whose intensities differ from the current pixel value are excluded from the filtering data. The filter achieves higher levels of PSNR with respect to other similar techniques such as 2D-LLMMSE, TA, and 3D-LLMMSE.

Jovanov et al. [27] use a motion estimation obtained in a video codec in a proposed video denoiser (WMVCE), introducing a motion field filtering step where the output of the same motion estimator is applied as an input for the coding scheme. In this filter, the temporal filtering precedes spatial filtering, and the remaining noise requires adaptivity of the spatial filter to the local noise statistics. This technique performs better than SEQWT and WST filters in terms of visual quality as well as in PSNR values for about 1 dB.

Melange et al. [36] report a fuzzy-logic-based filter (RFMDAF) focused on an additive white Gaussian noise model that improves the detail and motion adaptive multiple class averaging (MCA) filter. The method is first explained in the pixel domain for grayscale sequences and is later extended to the wavelet domain and to color videos. The proposed RFMDAF outperforms other state-of-the-art filters of comparable complexity when it is applied in different videos.

Kravchenko et al. [49] report a three-dimensional space-time filtering algorithm based on fuzzy sets theory (FDARTF_G). This denoising technique uses the gradient pixel values of the R, G, and B channels and the angular differences between pixels, filtering the neighboring frames on the basis of fuzzy logic rules. Simulation results evaluated via criteria of PSNR, MAE, and NCD have confirmed the superiority of this framework in comparison with VMMKNN and VGVDF_G filters.

Dabov et al. [50] present a video filtering method created on highly sparse signal representation in the local 3D transform domain (VBM3D). The 3D data array called “*group*” is performed by stacking together blocks found to be similar to the currently processed one. The formed 3D group is filtered by a 3D transform-domain shrinkage. The enhancement of this algorithm is reached by using a two-step algorithm where an intermediate estimate is produced by grouping and collaborative hard-thresholding and then used both for improving the grouping and for applying collaborative Wiener filtering.

The nonlocal means (NLM) filtering technique is employed for denoising of still images corrupted by additive noise and it uses all the possible self-predictions and self-similarities that the image can provide to determine the pixel weights for filtering the noisy image. Several filtering algorithms are reported based on this technique [51–53].

Other algorithms for filtering still images or video sequences have been developed in recent years [28, 32, 54–60].

Generally, the principal objective of different approaches to denoising mixed noises (additive and impulsive) consists of the detection and suppression of random spikes followed by filtering out additive noise [61, 62].

The filtering scheme proposed in this paper is based on fuzzy sets. A special characteristic of fuzzy filters is their self-skill to adapt based on local image data. The main purpose of a fuzzy filter is to remove noise from pixels that have been corrupted while preserving edges, fine features, textures, and chromaticity characteristics. For noisy pixels, the output of the filter is a specially selected pixel or is the result of the filtering method, which applies fuzzy rules to pixels located at the neighborhood of the contaminated one. Along the processing of neighboring frames, it is necessary that the filter has the ability to distinguish between possible local motions in the objects, fine details, and corrupted pixels. Fuzzy sets are a generalization of classical sets. While classical sets over a universe *X* can be characterized using *X* to {0,1} mappings, fuzzy sets are modeled by *X* to [0,1] mappings (membership functions). An element *x* ∈ *X* is or is not a member of a set in classical set theory. But in fuzzy set theory, a more gradual transition between membership and nonmembership is permitted; the degree of membership is between 0 and 1. For that reason, fuzzy sets are useful for processing human knowledge where linguistic variables (e.g., large, small, etc.) are used. For instance, a difference in grey level can be estimated as “LARGE,” “NO LARGE,” or “LARGE” to some level.

Fuzzy rules are linguistic *IF-THEN* constructions that have the general form “*IF A, THEN B*,” where *A* and *B* are the collections of propositions containing linguistic variables. The *IF* component of the rule, *A*, is called the premise or antecedent, and *B* is the consequence of the rule. A fuzzy membership function defines how each point in the input space is mapped to a membership value (or degree of membership) between 0 and 1. The membership function must vary between 0 and 1. The function can take any form and is defined by the programmer from its own point of view of efficiency, convenience, and simplicity.

Fuzzy filters are based on the observation that noise causes a small fuzzy derivative, whereas a large fuzzy derivative is caused by the presence of fine details or edges. Fuzzy rules can be applied in several directions and consider changes that can occur, such as local variations and variations in edges and fine features. In image filtering, fuzzy rules are able to distinguish between noisy pixels, edges, fine image features, and smoothed areas. These distinctions allow the main characteristics of an image to remain unchanged. In color videos, interchannel processing and motion detection algorithms are used to preserve fine details and edges, and only corrupted pixels should be filtered.

The *Fuzzy-Multichannel-Additive-Noise-Suppression* (FMANS 3D) filter is designed to suppress additive noise that corrupts images and color videos while preserving images features such as edges, chromaticity characteristics, texture, and fine details. Unlike other state-of-the-art methods, this novel technique consists of three principal filtering steps: spatial, spatiotemporal, and spatial postprocessing. In the first spatial step, the eight gradient values in different directions for pixels located in the vicinity of a central one as well as the R, G, and B channel correlation between analogous pixels are used, where the degree of contamination is estimated by employing novel fuzzy rules that allow for better preservation of image features. In the second spatiotemporal filtering step, two video neighboring frames are analyzed together, where the calculated similarity measure between pixels in the neighboring frames allows finding an interframe sample of the most similar pixels. Possible local motions between consecutive frames are estimated using a block matching procedure in different directions to finally perform interframe filtering. In the final stage, the spatial postprocessing filtering stage, the edge and smoothed regions of a current frame are distinguished using different filtering procedures. Numerous simulation results obtained for color videos with different texture characteristics, edges, color properties, and local motions have confirmed the superiority of this novel 3D fuzzy framework over other filtering techniques in terms of objective criteria (PSNR, MAE, NCD, and SSIM) as well as the subjective perception via human visual system. Additionally, the novel framework uses only two video frames concurrently, which reduces the computational processing time and memory requirements.

This paper is structured as follows: in Section 2 the proposed filter is explained; Section 3 presents and analyzes the simulation results and shows the performance evaluation and the experimental results are discussed; in Section 4 conclusions are presented.

## 2. Fuzzy Video Color Filter

The design of the *FMANS 3D* filter is divided into three stages: spatial, spatiotemporal, and spatial postprocessing steps. In the first spatial step, a single video frame is processed. The basic and six related gradient values existing between a central pixel located in a sliding window with respect to the pixels located in its vicinity are calculated. Applying designed fuzzy rules, the “*Fuzzy Similarity*” value is computed, and if this similarity is considered “*LARGE*,” a *weighted mean fuzzy filter* is used with the weights determined from introduced fuzzy rules, ending the current filtering step in the output denoted by ${\hat{E}}_{t}^{\eta }{\left(i,j\right)}_{1}$. In the opposite case, if the “*Fuzzy Similarity*” is considered “*NO LARGE*,” the fuzzy similarities and fuzzy weights are performed by taking into account the information in each specific color channel (R, G, or B) and employing the correlation between them. Lastly, in this step, weighted mean fuzzy filtering is executed with fuzzy weights computed from similarity measures for each channel, which include the channel and interchannel pixel properties, finally forming the output ${\hat{E}}_{t}^{\eta }{\left(i,j\right)}_{2}$.

In the second step, the spatiotemporal procedure is performed. Two neighboring frames are analyzed concurrently based on the difference between current (*t*) and previous (*t* − 1) frames. The “Fuzzy Similarity” set that allows the estimation of interframe changes. If this similarity is considered “LARGE,” then a weighted mean fuzzy filter is applied for a common sample that consists of the pixels in the analogous sliding windows from the current (*t*) and previous (*t* − 1) frames according to designed fuzzy rules that determine the weights for filtering, finally forming the output ${\hat{\hat{E}}}_{t}^{\eta }{\left(i,j\right)}_{1}$ of this part of the step. In the opposite case, when the *fuzzy similarity* is considered to be “*NO LARGE*,” the developed filtering scheme involves a block matching [50, 63] procedure that estimates the possible local motions between neighboring (*t*) and (*t* − 1) frames in the vicinity of a pixel in a sliding window. The block matching procedure is performed by calculating the differences between the pixel values located in the sliding window of a current frame (*t*) and an analogous moving (in possible eight directions) sliding window in the previous frame (*t* − 1). In the case of successful local motion estimation, when the “*Fuzzy Motion Similarity*” is “LARGE,” the fuzzy Alfa trimmed mean (*Alfa-TM*) filtering method [13] with fuzzy weights for common samples consisting of the pixels in the sliding window from the current frame (*t*) and moved sliding window from previous (*t* − 1) frames is used, yielding the output ${\hat{\hat{E}}}_{t}^{\eta }{\left(i,j\right)}_{2}$.

In the third step, if “*Fuzzy Motion Similarity*” is “*NO LARGE*,” considering that it is not possible to estimate well the local motion between groups of pixels in neighboring frames, an additional postprocessing procedure is performed: detection of the edges and their separation from smoothed regions. The fuzzy set “*Edge Detection Similarity*” is introduced using only the information from the actual frame (*t*). Therefore, if the “*Edge Detection Similarity*” is considered “*LARGE*,” then the *Alfa-TM* fuzzy weighting filtering in frame (*t*) is executed with weights based on computed fuzzy similarities, yielding the output ${\hat{\hat{E}}}_{t}^{\eta }{\left(i,j\right)}_{3}$. In the opposite case, when the plain areas are detected and “*Edge Detection Similarity*” is considered “*NO LARGE*,” the weighted mean fuzzy filter in frame (*t*) is applied with weights based on fuzzy similarities, yielding the output ${\hat{\hat{E}}}_{t}^{\eta }{\left(i,j\right)}_{4}$.

**Figure 1:**
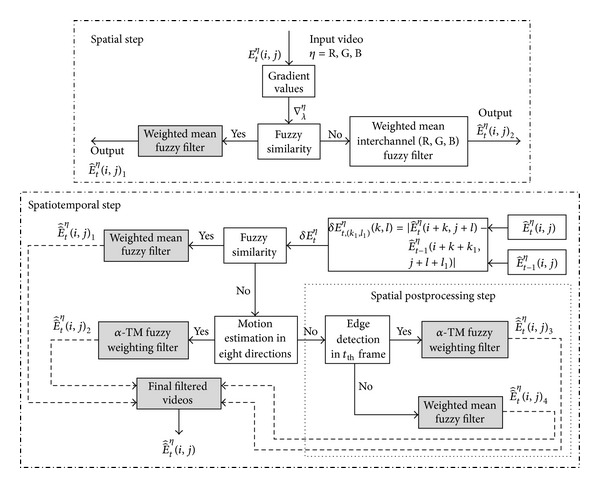
The general denoising scheme of the proposed filtering technique.

Figure 1 presents the details of the procedures employed in the proposed filter.

### 2.1. First Stage: Spatial Filtering

**Figure 2:**
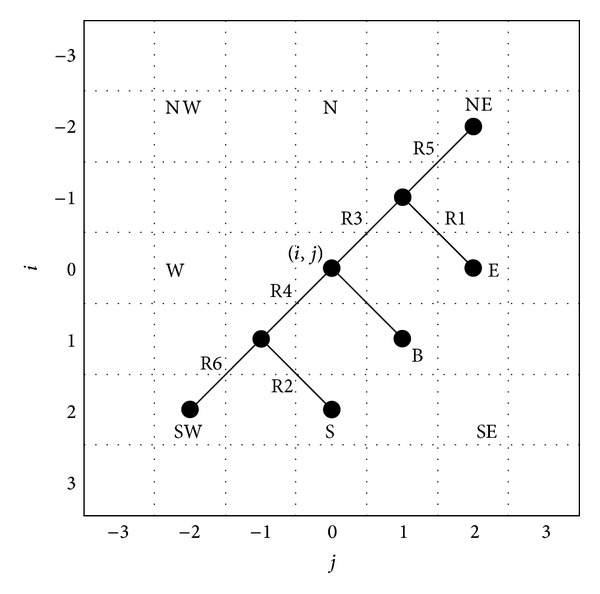
Six related and basic gradients.

The spatial filtering stage is executed as follows. Consider only the red (R) component because the procedure is similar for the green (G) and blue (B) bands. In the filtering procedure, a 5 × 5 sliding window at the center of a larger 7 × 7 sliding window is considered as shown in Figure 2. The gradient values in eight different directions are calculated as the absolute differences between the central pixel, defined as *E*_*c*_^*η*^ in the position (*i*, *j*) = (0,0), and the other pixels located along the eight directions in the neighborhood of the central pixel, defined as *E*_*λ*_^*η*^, where *λ* = {N, E, S, W, NW, NE, SE, SW}. Such differences are denoted by ∇_(*k*,*l*)_^*η*^ = |*E*_*c*_^*η*^(*i*, *j*) − *E* ^*η*^(*i* + *k*, *j* + *l*)|. The parameter *η* defines the chosen color channel in the RGB color space. For any direction, the basic gradient value and the six related gradient values are described by (*k*, *l*) values in the range {−3, −2, −1,0, 1,2, 3}. The related gradients are introduced to avoid spreading edges and fine features. Gradient values are employed to determine the degree of noise (from the impulsive point of view) and a level of “noise” for a central pixel, because in the presence of additive noise it is assumed that all the pixels have been contaminated. In this step, one basic gradient denoted by “B” and six related gradients denoted by “R1,” “R2,” “R3,” “R4,” “R5,” and “R6” are employed (see Figure 2) [43, 64]. Once we have computed the gradient values in the 7 × 7 sliding window, it is possible to find the degree of corruption for a central pixel in the “*Fuzzy Similarity*” set.

To estimate the noise contamination in the central pixel of a 7 × 7 sliding window, we introduce the “*LARGE*” and “*NO LARGE*” fuzzy sets in the following equations. A large membership degree (i.e., close to 1) in the fuzzy set “*NO LARGE*” indicates that the central pixel has sufficiently small level of noise contamination (noise), whereas, in the opposite case, a large membership degree in the fuzzy set “*LARGE*” indicates that the central pixel has a large degree of corruption introduced by noise. Membership functions can be designed from basic functions, for example, piecewise linear functions, a sigmoid function, quadratic and cubic polynomials, or the Gaussian function. Because of their simplicity and convenience, we use Gaussian membership functions [43, 49] to compute the membership degrees of fuzzy gradient values:

(1) $$\begin{array}{c} \tau \left(\nabla_{\lambda }^{\eta },\text{LARGE}\right)=\{\begin{array}{cc} 1, & \nabla_{\lambda }^{\eta }>\nabla_{1},\\ \exp-\left[\frac{{\left(\nabla_{\lambda }^{\eta }-\nabla_{1}\right)}^{2}}{2\delta^{2}}\right], & \text{otherwise}, \end{array} \end{array}$$

(2) $$\begin{array}{c} \tau \left(\nabla_{\lambda }^{\eta },\text{NO}\;\text{LARGE}\right)=\{\begin{array}{cc} 1, & \nabla_{\lambda }^{\eta }<\nabla_{2},\\ \exp-\left[\frac{{\left(\nabla_{\lambda }^{\eta }-\nabla_{2}\right)}^{2}}{2\delta^{2}}\right], & \text{otherwise}. \end{array} \end{array}$$

The values of the parameters used in (1) and (2) were determined based on the optimal values of the PSNR and MAE criteria (see Section 3.2). Fuzzy Rules 1 and 2 are proposed to resolve the following hypothesis: a central pixel belongs to an image detail or it has a large degree of corruption that determines how noisy it is.

In addition, the basic gradient “B” and the six related gradients “R1,” “R2,” “R3,” “R4,” “R5,” and “R6” are employed to avoid image blur detecting the presence of either plain areas or edges (Figure 2). In this case, the related gradient values are computed in the same direction as shown in (Figure 2), specifically, the SE direction fixed for the basic gradient. Thus, for the SE direction, where (*k*, *l*) = (−1, 1), the basic gradient value is ∇_*λ*_^*η*^ *E*_B_, and the six related gradient values are ∇_*λ*_^*η*^ *E*_R1_, ∇_*λ*_^*η*^ *E*_R2_, ∇_*λ*_^*η*^ *E*_R3_, ∇_*λ*_^*η*^ *E*_R4_, ∇_*λ*_^*η*^ *E*_R5_, and ∇_*λ*_^*η*^ *E*_R6_. If these gradient values are “*NO LARGE*” in the “*Fuzzy Similarity*” set, then the pixel (*i*, *j*) is considered to be corrupted.

The first *fuzzy rule* (see Table 1) defines the fuzzy gradient value ∇_*λ*_^*ηF*^ for a specific direction *λ*, which is contained in the “*Fuzzy Similarity*” set “*LARGE*.” The central pixel of the color channel is considered highly noisy if its basic “B” gradient value differs from the six related gradient values “R1,” “R2,” “R3,” “R4,” “R5,” and “R6” (Figure 2).

In this case, the logic operations “AND” and “OR” are defined as the following algebraic operations: AND = *A* · *B* and OR = *A* + *B* − *A* · *B*.

If a central pixel is considered to be noisy, it is necessary to determine how “*noisy*” it is.

Using Fuzzy Rule 2, the noisy factor Γ_*η*_ is obtained from the fuzzy gradient values in Fuzzy Rule 1 for each direction *λ* (see Table 1).

The noisy factor, Γ_*η*_, is computed gathering information from eight directions and is employed as a measure to distinguish between a highly *noisy* and a *low noisy* pixel, determining the amount of noise present in a central pixel of the given color component in the “*Fuzzy Similarity*” set “LARGE.” We present the experimental justification for this threshold in accordance with the optimal values of MAE and PSNR criteria (see Section 3.2). If the level of noise is lower than a certain threshold (Γ_*η*_ ≤ Th_1_), then the pixel should be filtered by employing a weighted mean fuzzy filter with the determined fuzzy weights, forming the output ${\hat{E}}_{t}^{\eta }{\left(i,j\right)}_{1}$ (Figure 1). In the filtering procedure, the fuzzy gradient values of the corrupted pixels are used as weights.

If a pixel is strongly corrupted when the degree of noise in a central pixel is higher than the threshold (Γ_*η*_ ≥ Th_1_), we propose the application of additional filtering to each channel datum by employing the existing interchannel correlations between the R, G, and B channel components.

A correlation between the R, G, and B channels arises if a certain number of local differences coincide with neighboring channel components.

Using membership functions from (1) and (2), it is possible to obtain the fuzzy similarities degrees *τ* ^R^, *τ* ^G^, and *τ* ^B^, which represent the differences between the other channels components and the values of neighboring color components.

The main idea of this procedure is based on the existence of two different relationships among the central component and its neighboring color components. Two different tasks are determined through this procedure: (a) the quantity of similarities between the central pixel and its neighbors in the same color band and (b) the quantity of similarities between the central window pixel in a certain color band and other pixels located in the same position but in a different color band.

The pixel located at the center of the sliding 3 × 3 window is defined as *E*_*C*_ = (*E*_*C*_^R^, *E*_*C*_^G^, *E*_*C*_^B^), and each pixel located in the vicinity of the same window is defined as *E*_*i*_ = (*E*_*i*_^R^, *E*_*i*_^G^, *E*_*i*_^B^); in addition, the neighboring pixels of the central pixel *E*_*C*_ are *E*_1_, *E*_2_,…, *E*_8_.

To calculate the absolute difference between the central component *E*_0_ and its neighbors, the following procedures are carried out [43]:

(3) $$\begin{array}{c} \Delta E_{\lambda }^{\text{R}}=\left|E_{C}^{\text{R}}-E_{\lambda }^{\text{R}}\right|,\;\;\Delta E_{\lambda }^{\text{G}}=\left|E_{C}^{\text{G}}-E_{\lambda }^{\text{G}}\right|,\\ \Delta E_{\lambda }^{\text{B}}=\left|E_{C}^{\text{B}}-E_{\lambda }^{\text{B}}\right|, \end{array}$$

where *λ* = 1,…, 8 and Δ*E*_*λ*_^R^, Δ*E*_*λ*_^G^, and Δ*E*_*λ*_^B^ represent the differences for the R, G, and B components, respectively.

The membership degree in the fuzzy set “*NO LARGE*” is established according to the desired behavior; that is, a relatively small difference is characterized by a “*LARGE*” membership degree. We use the same Gaussian membership function (2). The values of the parameters ∇_2,inter_ = 9 and *δ*_2,inter_^2^ = 750 employed in membership function (2) at this filtering stage have been chosen experimentally from numerous simulations according to the optimal values of PSNR and MAE criteria.

The membership degrees obtained, *τ*(Δ*E*_*λ*_^R^), *τ*(Δ*E*_*λ*_^G^), and *τ*(Δ*E*_*λ*_^B^), in the fuzzy set “*NO LARGE*” are used to determine if *E*_*C*_^R^, *E*_*C*_^G^, and *E*_*C*_^B^ are similar to their neighbors. Let us consider the red component only; the procedures for the G and B color bands are analogous. To ensure that the most relevant differences are considered, the *τ*(Δ*E*_*λ*_^R^) measures are ranked in descending order. Using (2), the similarities between *E*_*C*_^R^ and the closest neighbors are

(4) $$\begin{array}{c} \tau_{\lambda }^{\text{R}}=\prod_{j=1}^{Q}\tau \left(\Delta E_{\left(j\right)}^{\text{R}}\right). \end{array}$$

Then, the similarity measures between pixels in the selected color channel and the corresponding pixels from the other two color bands are calculated, that is, |*τ*(Δ*E*_*λ*_^R^) − *τ*(Δ*E*_*λ*_^G^)| and |*τ*(Δ*E*_*λ*_^R^) − *τ*(Δ*E*_*λ*_^B^)|. In order to compute this, the membership degree of the next fuzzy set is obtained using the chosen parameter values ∇_2,*η*_1_*η*_2__ = 0.004 and *δ*_2,*η*_1_*η*_2__^2^ = 0.03 determined experimentally:

(5) $$\begin{array}{c} \tau_{\lambda }^{\text{RG}}=\tau_{1}\left(\left|\tau \left(\Delta E_{\lambda }^{\text{R}}\right)-\tau \left(\Delta E_{\lambda }^{\text{G}}\right)\right|\right),\\ \tau_{\lambda }^{\text{RB}}=\tau_{1}\left(\left|\tau \left(\Delta E_{\lambda }^{\text{R}}\right)-\tau \left(\Delta E_{\lambda }^{\text{B}}\right)\right|\right). \end{array}$$

The membership degrees *τ*_*λ*_^RG^ and *τ*_*λ*_^RB^ indicate whether the local difference between the center pixel and the pixel in position *λ* in the R component is similar to the local differences in the G and B components. The computed *τ*_*λ*_^RG^ and *τ*_*λ*_^RB^ are ranked in descending order, and the similarity measure is calculated as follows:

(6) $$\begin{array}{c} \tau^{\text{RG}}=\prod_{j=1}^{Q}\tau_{\left(j\right)}^{\text{RG}},\;\;\tau^{\text{RB}}=\prod_{j=1}^{Q}\tau_{\left(j\right)}^{\text{RB}}. \end{array}$$

Next, Fuzzy Rule 3 defines the condition in which the R component pixel can be characterized as exhibiting a low level of noise (see Table 1).

The values of the parameters used in membership function (2) during the interchannel filtering step were selected according to the best values obtained for the PSNR and MAE criteria after numerous simulations ∇_2,inter_ = 9, *δ*_inter_^2^ = 400, ∇_2,*λ*_1_*λ*_2__ = 0.004, and *δ*_*λ*_1_*λ*_2__^2^ = 0.03 (see Section 3.2).

After computing Fuzzy Rule 3, the fuzzy weights in the fuzzy set “Noise-free” are obtained as follows:

(7) $$\begin{array}{c} W\left(E_{\kappa ,\text{free}}^{\text{R}}\right)=\tau^{\text{R}}\tau^{\text{RG}}\tau^{\text{G}}+\tau^{\text{R}}\tau^{\text{RB}}\tau^{\text{B}}-\tau^{\text{R}}\tau^{\text{RG}}\tau^{\text{G}}\tau^{\text{R}}\tau^{\text{RB}}\tau^{\text{B}},\\ W\left(E_{\kappa ,\text{free}}^{\text{B}}\right)=\tau^{\text{B}}\tau^{\text{BR}}\tau^{\text{R}}+\tau^{\text{B}}\tau^{\text{BG}}\tau^{\text{G}}-\tau^{\text{B}}\tau^{\text{BR}}\tau^{\text{R}}\tau^{\text{B}}\tau^{\text{BG}}\tau^{\text{G}},\\ W\left(E_{\kappa ,\text{free}}^{\text{G}}\right)=\tau^{\text{G}}\tau^{\text{GR}}\tau^{\text{R}}+\tau^{\text{G}}\tau^{\text{GB}}\tau^{\text{B}}-\tau^{\text{G}}\tau^{\text{GR}}\tau^{\text{R}}\tau^{\text{G}}\tau^{\text{GB}}\tau^{\text{B}}. \end{array}$$

Fuzzy Rules 4 and 5 are developed to compute the fuzzy weights in the filtering procedure for the central pixel in its color band R as well as the same pixel with respect to the other color bands (see Table 1).

Similar fuzzy rules should be applied for the other two channels:

(8) WEλR=NECRNEEλRτ(ΔEλG)NEEλG+NECRNEEλRτ(ΔEλB)NEEλB −NECRNEEλRτ(ΔEλG)NEEλG ·NECRNEEλRτ(ΔEλB)NEEλB.

These fuzzy weights are used in this step of the noise suppression algorithm, where the weights determined according to *Fuzzy Rule *5 and (5) are used in the mean filtering procedure:

(9) $$\begin{array}{c} {\hat{E}}_{t}^{\eta }=\frac{\sum_{\lambda }^{}W_{E_{\lambda }^{\eta }}E_{\lambda }^{\eta }}{\sum_{\lambda }W_{E_{\lambda }^{\eta }}}. \end{array}$$

### 2.2. Second Stage: Spatiotemporal Filtering

In this step, two neighboring frames of a video are processed together. The results of the spatial filtering in frame (*t*) denoted by ${\hat{E}}_{t}^{\eta }(i,j)$ and the analogous results from spatial filtering in frame (*t* − 1) obtained via the procedure described in the previous section denoted by ${\hat{E}}_{t-1}^{\eta }(i,j)$ are applied (see Figure 1).

The differences between the (*t*) and (*t* − 1) frames are calculated as follows:

(10) δEt,(k1,l1)η(k,l) =|E^tη(i+k,j+l)−E^t−1η(i+k+k1,j+l+l1)|,

where *δE*_*t*,(*k*_1_,*l*_1_)_^*η*^(*k*, *l*) denotes the frame difference and (*k*, *l*)∈{−3, −2, −1,0, 1,2, 3}; (*k*_1_, *l*_1_)∈{−1,0, 1}.

The Gaussian membership functions presented in (1) and (2) are employed using parameters that have been adjusted for the difference frame, *δE*_*t*_^*η*^(*k*, *l*), in the “*Fuzzy Similarity*” set according to the optimal values of the PSNR and MAE criteria.

A procedure similar to that carried out in the spatial step is now executed in the spatiotemporal step. It is necessary to determine how noisy the difference pixel is, using the “B,” “R1,” “R2,” “R3,” “R4,” “R5,” and “R6” gradient values as shown in Figure 2 (case for nonmoving frames, *k*_1_ = *l*_1_ = 0). These values should be employed if the gradient value is considered to be “*LARGE*” in the “*Fuzzy Similarity*” set used to analyze a new measure: a second fuzzy noisy factor denoted by *ε*_*η*_, where *ε*_*η*_ ≤ Th_2_ (see Figure 1).

Fuzzy Rule 6 is employed to determine if the fuzzy gradient value is considered to be “*LARGE*” in the “*Fuzzy Similarity*” set (see Table 1).

Fuzzy Rule 7 defines the fuzzy noisy factor *ε*_*η*_ (see Table 1).

Assuming that the “*Fuzzy Similarity*” is “*LARGE*,” when *ε*_*η*_ ≤ Th_2_, where Th_2_ = 0.45 (see Section 3.2), then a fuzzy weighted mean fuzzy filter is applied for the common sample that includes the pixels from both analogous sliding windows from frames (*t*) and (*t* − 1) according to the fuzzy measures, thus yielding the output ${\hat{\hat{E}}}_{t}^{\eta }{\left(i,j\right)}_{1}$.

In the opposite case, when the “*Fuzzy Similarity*” is considered to be “*NO LARGE*,” it is necessary to estimate the possible motion between frames (*t*) and (*t* − 1) by analyzing the directions *λ*. The developed filtering scheme involves a block matching procedure that estimates the local motion between neighboring frames (*t*) and (*t* − 1).

Let us introduce the parameter *ρ*_*η*_, which characterizes the estimated local motion and the threshold Th_3_ in this case. When *ρ*_*η*_ ≤ Th_3_ occurs, the local motion between the analyzed parts of the (*t*) and (*t* − 1) frames should be considered. In this case, it is necessary to compute the difference between neighbors of the central pixel in frame (*t*) and the pixels located at the analogous positions in the previous frame (testing the eight possible directions *λ*) to determine the most similar parts among the pixels in the current and previous frames.

A global motion model does not reflect local interframe motions. Block matching is a standard procedure used in video processing to compensate for local interframe motions. In this procedure, each of the analogous pixels in a sliding window in the neighboring frames is replaced by a version that is formed from the pixels in the motion-compensated window that coincides with the reference frame. The compensated part of the frame is then established by pasting the best matching block of the neighboring previous frame to the position of the block in the current reference.

In this study, we use the measure of similarity between blocks in form of the *mean of the absolute difference* (MAD). Moreover, rather than attempting to find the best matches for every block of the reference frame, we consider only blocks where significant motion has occurred because in highly noisy videos there is a strong risk of matching the noise component in smoothed regions. In that case, the interframe noise becomes locally highly correlated.

The criterion MAD that we use to find the highest degree of similarity between the central pixel in the sliding window in frame (*t*) and the analogous window in frame (*t* − 1) [63] is defined as follows:

(11) $$\begin{array}{c} {\text{SAD}}_{\left(i,j\right)}E_{\left(k_{1},l_{1}\right)}=\sum_{k=0}^{M-1}\;‍\sum_{l=0}^{N-1}C_{kl}\delta E_{t,\left(k_{1},l_{1}\right)}^{\eta }\left(k,l\right). \end{array}$$

We therefore should ultimately determine the best match minimizing SAD_(*i*,*j*)_ *E*_(*k*_1_,*l*_1_)_ for the eight directions analyzed, *λ* = {N, E, S, W, NW, NE, SE, SW}, varying parameters *k*_1_, *l*_1_. In simulation experiments, we observed that better results can be obtained if the weight *C*_00_ for a central pixel is significantly larger than that of other pixels. Thus, the criterion for possible motion in areas surrounding a central pixel (*i*, *j*) in frame (*t*) is written as follows:

(12) $$\begin{array}{c} \rho_{\eta }=\min_{\left\{k_{1},l_{1}\right\}}\text{MAD}\delta E_{t,k_{1},l_{1}}^{\eta }. \end{array}$$

This motion estimation should be taken into account if and only if the minimum MAD determined is less than threshold Th_3_:

(13) $$\begin{array}{c} \rho_{\eta }\leq {\text{Th}}_{3}. \end{array}$$

In the case of successful local motion estimation, when the “*Fuzzy Motion Similarity*” set is “*LARGE*,” the *Alfa-TM* filtering with weights based on fuzzy similarities is used for a common sample consisting of the pixels in the sliding window from current frame (*t*) and the moved sliding window from the previous frame (*t* − 1). In this step, the fuzzy gradient values computed in both frames are used to obtain the fuzzy weights, where in the fuzzy *Alfa-TM* filter the most similar neighbors are taken into account, eliminating other outermost neighbors as defined in the filter equation. The output for this procedure is defined as ${\hat{\hat{E}}}_{t}^{\eta }{\left(i,j\right)}_{2}$.

Fuzzy Rule 8 is employed to determine the fuzzy weights of the red component *WE*_*η*_^R^ in the fuzzy *Alfa-TM* fuzzy filter (see Table 1):

(14) $$\begin{array}{c} {\hat{\hat{E}}}_{t}^{\eta }=\frac{\sum_{\lambda \in [a,2N-a]}^{}W_{E_{\lambda }^{\eta }}E_{\lambda }^{\eta }}{\sum_{\lambda \in [a,2N-a]}W_{E_{\lambda }^{\eta }}}, \end{array}$$

where *N* is the number of pixels to be processed from the frames (*t*) and (*t* − 1).

### 2.3. Third Stage: Spatial Postprocessing Filtering

When the “*Motion Fuzzy Similarity*” set is considered to be “*NO LARGE*” (*ρ*_*η*_ > Th_3_), it is difficult to perform motion compensation well. We cannot employ the information gathered from the previous frame (*t* − 1); only the data from the current frame is taken into account. At this point, we introduce the “*Edge Detection Similarity*” fuzzy set. Fuzzy Rule 9 then helps to determine the presence of edges or plain areas in the frame (see Table 1).

To find these similarities, let us compute the related gradient values “R1” and “R2,” which are used with gradient value “B” to determine the presence of plain areas in the analyzed frame (see Figure 2); in the opposite case, gradient values “R3,” “R4,” “R5,” “R6,” and “B” help to determine the presence of edges.

Considering the foregoing discussion, two different processes should be employed. If the “*Edge Detection Similarity*” is considered to be “*LARGE*,” then a fuzzy *Alfa-TM* filtering is executed in frame (*t*) based on fuzzy similarities according to fuzzy weights, as described in Fuzzy Rule 9 and (15).

Fuzzy Rule 10 defines the weights for the *Alfa-TM* filter *WE*_*λ*_^R^ in the case of edge detection (see Table 1):

(15) $$\begin{array}{c} {\hat{\hat{E}}}_{t}^{\eta }=\frac{\sum_{\lambda \in [a,N-a]}W_{E_{\lambda }^{\eta }}E_{\lambda }^{\eta }}{\sum_{\lambda \in [a,N-a]}W_{E_{\lambda }^{\eta }}}. \end{array}$$

The output for this procedure is defined as ${\hat{\hat{E}}}_{t}^{\eta }{\left(i,j\right)}_{3}$ (see Figure 1).

Lastly, for smoothed regions, where “*Edge Detection Similarity*” is considered to be “*NO LARGE*,” a weighted mean fuzzy filter in frame (*t*) is executed based on fuzzy similarities, where the output for this step is denoted by ${\hat{\hat{E}}}_{t}^{\eta }{\left(i,j\right)}_{4}$ (see Figure 1).

Fuzzy Rule 11 determines the weight for the mean filter in the fuzzy “Edge Detection Similarity” set (see Table 1).

In this step, the filtering is defined as an averaging procedure with weights as follows:

(16) $$\begin{array}{c} {\hat{\hat{E}}}_{t}^{\eta }=\frac{\sum_{\lambda }W_{E_{\lambda }^{\eta }}E_{\lambda }^{\eta }}{\sum_{\lambda }W_{E_{\lambda }^{\eta }}}. \end{array}$$

## 3. Performance Evaluation

### 3.1. Performance Criteria

In order to evaluate the effectiveness of the proposed filter in suppression of additive noise and image detail preservation, the novel filter has been compared with other known techniques. The filtered frames were evaluated according to the following objective criteria.

We have employed the PSNR (*peak signal-to-noise ratio*) used to characterize the noise suppression capabilities of the proposed technique and the MAE (*mean absolute error*) that measures the level of preservation of edges and fine details [1, 14, 64, 65]. These two metrics are defined in the RGB color space:

(17) $$\begin{array}{c} \text{PSNR}=10\;\log_{10}\left[\frac{{\left(255\right)}^{2}}{\text{MSE}}\right],\text{dB}, \end{array}$$

where the MSE is the *mean squared error.*

The MAE is defined as

(18) MAE=1MN×∑i=1M ∑j=1N[(|R(i,j)−Re(i,j)|+|G(i,j)−Ge(i,j)|     +|B(i,j)−Be(i,j)|)×(3)−1].

For both cases in the MAE and MSE criteria, R(*i*, *j*), G(*i*, *j*), and B(*i*, *j*) represent the RGB color components of the original frame. Meanwhile, R_*e*_(*i*, *j*), G_*e*_(*i*, *j*), and B_*e*_(*i*, *j*) represent the color RGB components at the output of the filtered frame.

The NCD (*normalized color difference*, defined in the L*u*v* color space) [1, 38, 64] is commonly used to measure color preservation. To calculate the NCD criteria, the image must be converted to the L*u*v* color space. The error between color vectors Δ*E*_*Luv*_ = [(ΔL*)^2^ + (Δu*)^2^ + (Δv*)^2^]^1/2^ is employed to calculate the NCD measure:

(19) $$\begin{array}{c} \text{NCD}=\frac{\sum_{i=1}^{M}\sum_{j=1}^{N}||\Delta E_{Luv}||}{\sum_{i=1}^{M}\sum_{j=1}^{N}||e_{Luv}^{*}||}, \end{array}$$

where *e*_Luv_* = [(L*)^2^ + (u*)^2^ + (v*)^2^]^1/2^ is the magnitude of the original image (uncorrupted) pixel vector in the L*u*v* color space and *M* and *N* are the image dimensions.

The standard quality metrics used in the past such as PSNR can be erroneous in some cases. Novel metrics such as SSIM (*similarity structural index measure*), which match better human subjectivity, are applied to characterize the performance of the algorithm. For monochrome images, the SSIM metric values are defined as follows [66, 67]:

(20) $$\begin{array}{c} {\text{SSIM}}_{\beta }\left(e,E_{\text{out}}\right)=\left[l_{\beta }\left(e,E_{\text{out}}\right)\right]\cdot \left[c_{\beta }\left(e,E_{\text{out}}\right)\right]\cdot \left[s_{\beta }\left(e,E_{\text{out}}\right)\right], \end{array}$$

where “*l*,” “*c*,” and “*s*” are calculated for each color channel.

Here *E*_out_ is the filtered image and “*e*” is the original (uncorrupted) image; the “*l*” represents the luminance similarity, “*c*” characterizes the contrast similarity, and, finally, “*s*” is the structural similarity for a chosen channel (R, G, or B). The justification of the SSIM index can be found in [43, 67].

The key idea behind the SSIM index is to recognize that natural images are highly structured and that the measure of structural correlation between the original (uncorrupted) and the filtered image is very important in deciding the overall visual quality. Further, the SSIM index measures quality locally and is able to capture local dissimilarities better. Finally, we calculate the mean value of this quality index:

(21) $$\begin{array}{c} \text{SSIM}=\frac{1}{3}\left[{\text{SSIM}}_{\text{R}}+{\text{SSIM}}_{\text{G}}+{\text{SSIM}}_{\text{B}}\right]. \end{array}$$

We also use a subjective visual perception presenting for different color video sequences the filtered frames and/or their error images for several better state-of-the art filters to compare the capabilities of noise suppression and detail preservation.

### 3.2. Parameter Selection

**Figure 3:**
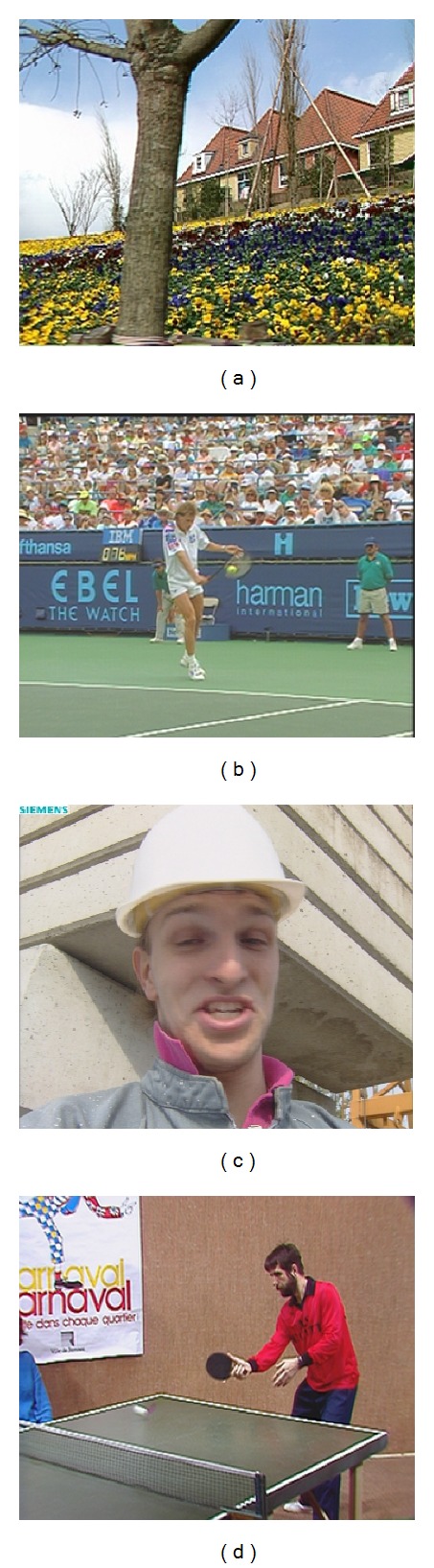
Original frames of the color video sequences: Flowers (20th), Stefan (20th), Foreman (80th), and Tennis (81st).

As mentioned previously, the membership functions presented in (1) and (2) are completely determined by their respective parameters. These parameter values, ∇_1_, ∇_2_, and *δ* ^2^, for different stages of the proposed algorithm have been experimentally optimized using the *Flowers*, *Stefan*, *Foreman*, and *Tennis* sequences (see Figure 3), which all have distinct characteristics. The Flowers video combines very detailed regions with strong edges, such as flower fields, houses, and trees, and homogeneous regions, such as sky backgrounds. The Stefan video features the very rapid motions of a tennis player over a tennis court with additional camera movements in the scene. The Foreman video features moderate head movement of the man against a detailed background that contains objects with strong edges. Lastly, in the Tennis sequence, we deal with the zooming camera and the wall as a detailed background, the lines in the table, and also the fast movements of the players.

The parameters were optimized as follows. The proposed method was applied to each of the previous sequences, which were contaminated in every single color channel with additive noise of different noise levels with variance *σ* ^2^ ranging from 0.0 to 0.030 and with membership function parameters varying over the entire range of possible values. After plotting the optimal parameter values in terms of PSNR and MAE criteria for the different sequences and noise levels, the optimal parameter values of the membership functions were determined. Therefore, the parameters have been determined by the best fit through observations of color sequences with different textures, motions, fine features, and color properties, confirming the robustness of the designed framework. Four different thresholds were employed in the proposed algorithm (Th_1_ = 0.22, Th_2_ = 0.45, Th_3_ = 0.37, and Th_4_ = 0.21) as well. The optimal values for the parameters are presented in Tables 2 and 3.

The following values were adopted: ∇_1_ = 75, ∇_2_ = 12, and *δ* ^2^ = 900 (Table 4). The parameters for the interchannel filtering stage ∇_2,*η*_1_*η*_2__ = 0.004 and *δ*_*η*_1_*η*_2__^2^ = 0.03 are shown in Table 5.

### 3.3. Efficiency of the Proposed Filter

In this section, several experiments were carried out to evaluate and compare the performance of the designed *FMANS 3D* technique with many state-of-the-art methods. Extensive experiments are conducted on different noisy color videos (*Flowers, Stefan, Foreman,* and *Tennis*) in the *CIF* format (352 × 288) (Figure 3) demonstrating the superiority of novel framework in the suppression of a noise and preserving the edges, fine details, and color chromaticity. The frames of the color videos were artificially contaminated by additive noise with different values of *σ* ^2^ (from 0 to 0.030) in each color band independently. These videos feature different textures, edge and fine details, chromaticity characteristics, and local motions that are varying from frame to frame.

To validate the superiority of the proposed method FMANS 3D, its performance is compared in terms of PSNR, MAE [1, 65], NCD [64], and SSIM [67] of the denoised images. The PSNR, MAE, and NCD are objective criteria measurements, whereas the SSIM better captures the human perception. These objective criteria and subjective perception via human vision system were used to test the performance of the designed filter over an average of 50 consecutive frames against other techniques, such as those developed by 3D-LLMMSE [26], WMVCE [27], RFMDAF [36], FDARTF_G [46], VBM3D [50], and NLM [52]. Mentioned filters were computed and used in accordance with their references comparing them with the proposed *FMANS 3D* framework. The reason for choosing these filters to compare with the designed technique is that their performances have been compared with various known video color filters, demonstrating their superiority in terms of objective and subjective criteria among all known filtering techniques.

**Figure 4:**
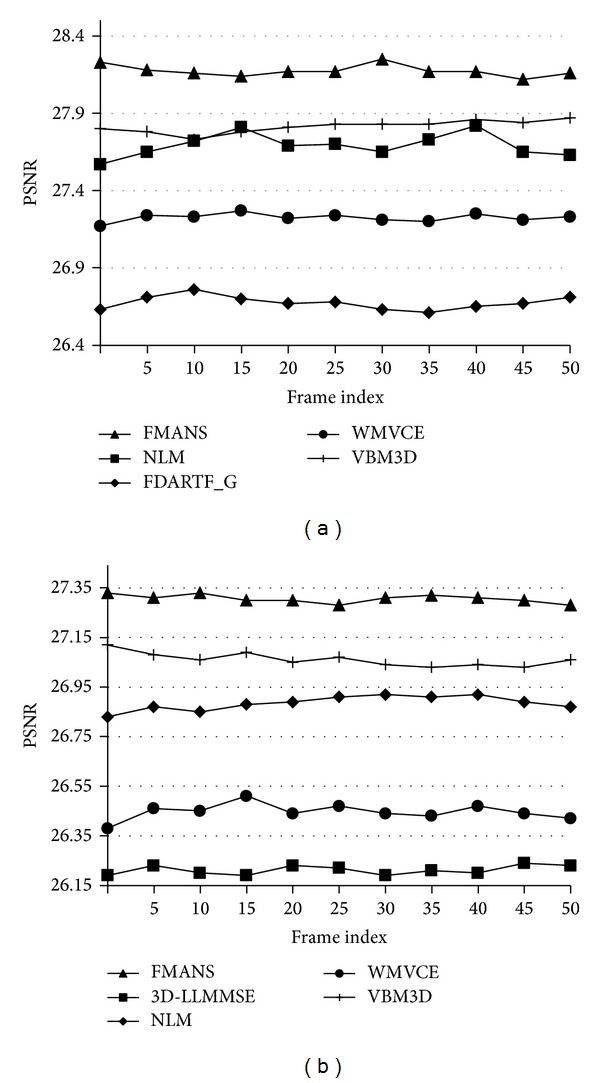
PSNR (dB) for the different methods applied to (a) *Flowers* (*σ* ^2^ = 0.005) and (b) *Tennis* (*σ* ^2^ = 0.015).

**Figure 5:**
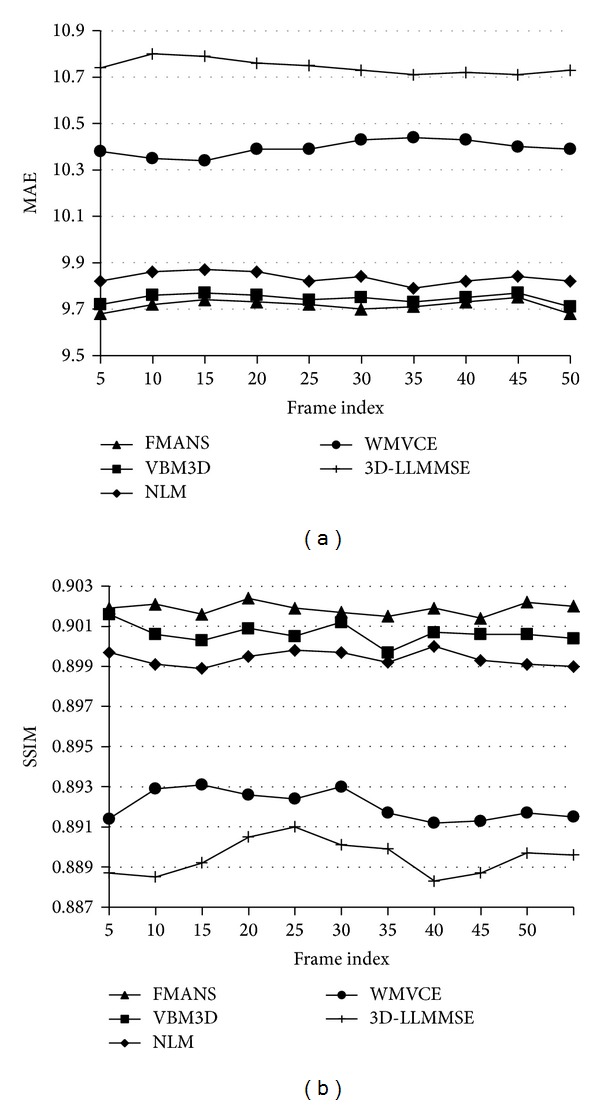
MAE for the different methods applied to (a) *Foreman* (*σ* ^2^ = 0.020) and SSIM for the different methods applied to (b) *Stefan* (*σ* ^2^ = 0.005).

Tables 6, 7, 8, and 9 demonstrate that our proposal outperforms better state-of-the-art techniques according to the objective criteria. The proposed algorithm and the other techniques indicated in the tables were evaluated in terms of the averaged PSNR, MAE, NCD, and SSIM criteria values applied to the four different color videos. As one can see, the designed *FMANS 3D* technique outperforms other analyzed filters in all the experiments for different color videos demonstrating the robustness in cases of varying texture, color properties, and local motions from frame to frame, exposing the best values for all objective criteria. The behavior of the filters' performance across the videos is illustrated in Figures 4 and 5 for 50 consecutive frames. Again, we can see stable superiority of novel filtering approach in objective criteria values, demonstrating better noise suppression, edge and fine details preservation, and chromaticity characteristics. Also, from these objective criteria values we can conclude that the designed technique performs essentially better than VBM3D algorithm.

**Figure 6:**
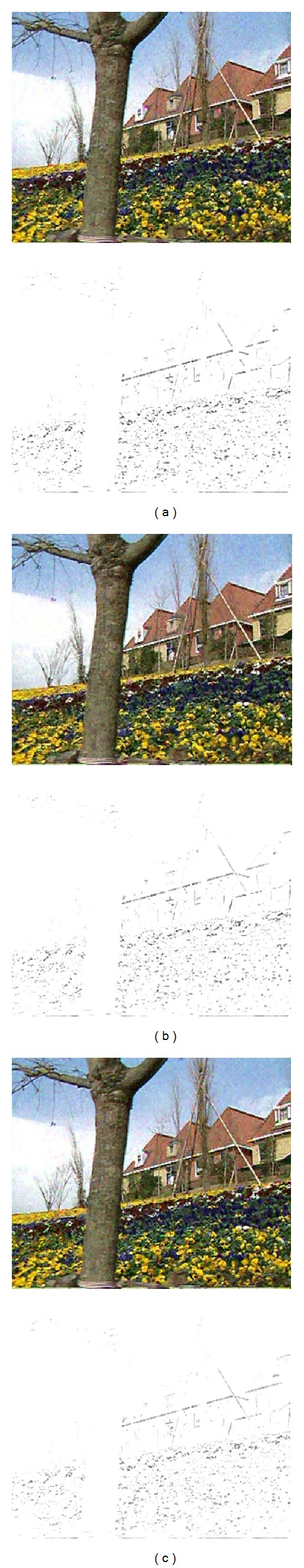
Filtered 20th frame of Flowers video (first row) and respective error images (second row) in case of *σ* ^2^ = 0.015 noise intensity of additive noise: NLM [52] (a), VBM3D [50] (b), and FMANS 3D (c) filters. The value of each error pixel is amplified 3 times in order to distinguish the details.

**Figure 7:**
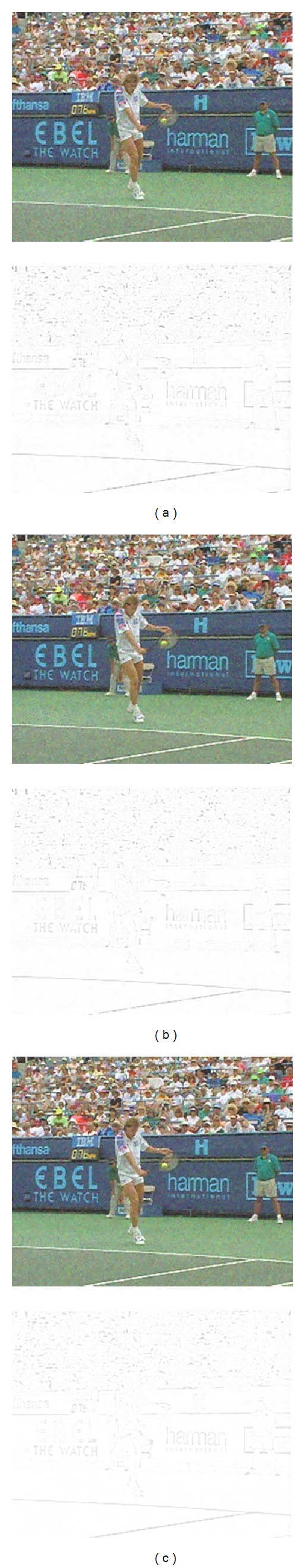
Filtered 20th frame of Stefan video (first row) and respective error images (second row) in case of *σ* ^2^ = 0.020 noise intensity of additive noise: NLM [52] (a), VBM3D [50] (b), and FMANS 3D (c) filters. The value of each error pixel is amplified 3 times in order to distinguish the details.

**Figure 8:**
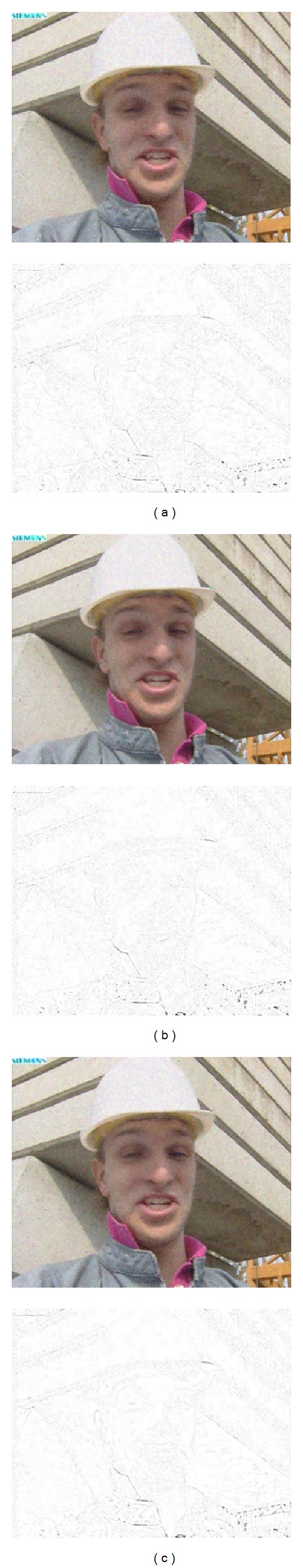
Filtered 80th frame of Foreman video (first row) and respective error images (second row) in case of *σ* ^2^ = 0.010 noise intensity of additive noise: NLM [52] (a), VBM3D [50] (b), and FMANS 3D (c) filters. The value of each error pixel is amplified 3 times in order to distinguish the details.

**Figure 9:**
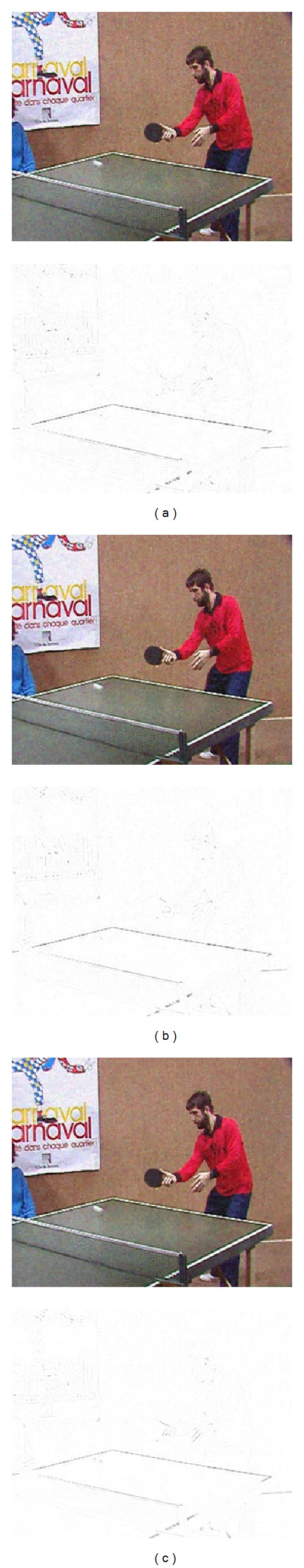
Filtered 81st frame of Tennis video (first row) and respective error images (second row) in case of *σ* ^2^ = 0.020 noise intensity of additive noise: NLM [52] (a), VBM3D [50] (b), and FMANS 3D (c) filters. The value of each error pixel is amplified 3 times in order to distinguish the details.

The visual quality of some of the restored images can be evaluated from Figures 6 to 9, where for a comparison we show results by the closest competitors *NLM* [52] and *VBM3D* [50]. The figures show the filtering frames and their error images for the 20th frame of Flowers (see Figure 6), where it is possible to appreciate that the proposed framework best preserves the leaves and details of the roofs of the houses. In the 20th frame of the Stefan video (see Figure 7), one can observe the better preservation of details in the field and letters located on the front wall compared with the other methods analyzed. Analyzing the 80th frame of the Foreman video (Figure 8) shows that fine details in the areas of eyes are better preserved using the proposed framework than when using the other filters. Lastly, in the 81st frame of the Tennis video (Figure 9), it is possible to appreciate by looking carefully that the texture and fine details on the wall are best preserved by our method as well as the preservation of the lines on the table. Overall, as indicated in Tables 6 to 9, one can see that the proposed algorithm is able to suppress the ringing artifacts and provides sharper image edges and objects are preserved better than *VBM3D* and the other techniques.

Other filters are based on a fixed-size 3D search neighborhood for the grouping by block matching technique: as a milestone in the research of image denoising, the *VBM3D* achieves remarkable results because it fully exploits the sparsity within a single image. In the preprocessing step in the *VBM3D*, the groups of similar patches within an image are formed into 3D patch cubes. Then, the 3D wavelet procedure is applied as mentioned in the Introduction. This method works well on image frames with abundant repetitive patterns. However, for video frames with unique patches (which have few similar patches in the image), the *VBM3D* produces suboptimal results. Our proposal as shown in simulation data outperforms the *VBM3D* results. For video sequences that present numerous plane areas such as Foreman on middle noise intensity (*σ* ^2^ = 0.003–0.01), the PSNR difference between the designed *FMANS 3D* and *VBM3D* filters is of about 0.25 dB (of about 5% in absolute PSNR values), and for high noise intensity (*σ* ^2^ > 0.015) the designed *FMANS 3D* filter increases slightly the difference with respect to the *VBM3D* filter of about 0.32 dB (of about 7.5% in absolute PSNR values). On the other hand, for video sequences that present different moving and changing areas where it is difficult to find similar objects in forming 3D patch cubes (Stefan and Flowers), our *FMANS 3D* filter seems to achieve better results: of about 0.4 dB (of about 10% in absolute PSNR values) in wide noise intensity range (0.000–0.03). Finally, for the Tennis video where the presence of fast moving subjects, zooming camera, and a detailed wall exist, the difference in the noise intensity range (0.000–0.005) is about 0.3 dB (of about 7% in absolute PSNR values) and for other noise intensity (*σ* ^2^ > 0.010) the *FMANS 3D* filter decreases slightly the difference with respect to the *VBM3D* filter of about 0.25 dB (5% in absolute PSNR values).

Some filtering techniques convert the RGB space into another color space such as L*a*b* or YCbCr color space since the human eye is far less sensitive to some details in chrominance or luminance; according to this, it is acceptable to filter only one component instead of three. Nevertheless, to achieve better results, we use the correlation among the three color bands since the spatial information existing in each one of them provides substantial information.

It is exposed from Figures 6 and 7 that the proposed *FMANS 3D* shows the best performance and it outperforms all of the other methods. In particular, a significant improvement is observed in the videos that contain structured details and smoothed areas such as Flowers (the leaves and details of the roofs of the houses) and Stefan (the public details in the field and letters located on the front wall).

Regarding the subjective visual quality, we find that various image details are well preserved and at the same time very few artifacts are introduced as one can observe this fact in Figures 6 to 9.

It is clear that the complexity of the proposed filter is linear in terms of the number of pixels in the frame. Every pixel is filtered by averaging a constant number of neighborhood pixels, which are all assigned a weight using a constant number of operations.

The proposed *FMANS 3D* framework provides the best results with significant advantage over the closest competitors. Particularly interesting is the comparison against the *VBM3D* algorithm.

As shown, the designed *FMANS 3D* combines sufficiently good detail preservation with good noise removal and appears to outperform other comparable filters. Another advantage is that the presented filtering method employs only two neighboring frames that, in rapidly moving regions, can facilitate spatiotemporal processing.

Our main contributions in this proposal are:

- employing novel fuzzy rules to select the group of the most similar pixels in the vicinity of estimating one via using the correlation in the color channels (R, G, and B);
- interframe processing employing the neighboring frames in a video together for better preservation of the features via adjusting the possible local motions;
- distinguishing and processing separately the areas that present different texture behavior (smoothed regions, edge, and fine features).

## 4. Conclusions

A novel 3D filtering framework FMANS for the denoising of color videos corrupted by additive noise is proposed.

The framework consists of three principal filtering steps: spatial, spatiotemporal, and spatial postprocessing filtering.

The proposed technique is based on fuzzy logic theory in combination with basic and several related gradient values along different directions, interchannel correlations, and employs the previous and current temporal frames. In the spatiotemporal filtering step, two neighboring frames are processed together, where the possible local motions between neighboring frames are estimated, thus increasing the filtering performance. In the postprocessing step, the edge and smoothed regions are distinguished and denoised differently, allowing for better filtering quality to be obtained. Based on the experimental results shown in the previous section for the PSNR, MAE, NCD, and SSIM criteria and a perception analysis via human visual system in the filtered videos, the novel approach was observed to be extremely efficient in reproducing the chromatic characteristics of images. We have demonstrated that this novel framework exhibits better processing performance than the best fuzzy and nonfuzzy filters for color videos with varying texture characteristics, edges, color properties, and local motions, successfully suppressing additive noise over a wide range of intensities and preserving edges and fine image features, demonstrating sufficiently good robustness.

Future work should be performed to improve the current method by incorporating more information to better distinguish between the level of noise in pixels, fine image features, and local motions, designing other fuzzy rules. We believe that the proposed denoising method could be improved by using more sophisticated frameworks, for example, via combining the current fuzzy approach with *Wiener* collaborating filtering [58] as a next filtering step. Next works will be focused on adaptation of fuzzy-based approach in the processing of the noisy videos under more complex noise models such as additive colored noise and non-Gaussian noise, by modifying the calculation of membership function parameters. Additional efforts will be made in implementing the proposed technique on a DSP platform by *Texas Instruments* (model TMS320DM648) and in the parallel processing hardware (GPU model QUADRO K2000d by *NVIDIA*) where an analysis of the processing speeds of better 3D algorithms and computation times will be reported.

However, the computational complexity would significantly increase because these transforms are typically nonseparable and do not have fast algorithms.

## Figures and Tables

**Table 1:** Fuzzy rules used in the FMANS 3D filter.

**Fuzzy Rule 1**. Defining the fuzzy gradient value ∇_λ_^ηF^ into the fuzzy similarity set LARGE	**IF** ((∇_λ_^η^B is LARGE AND ∇_λ_^η^R1 is LARGE) OR (∇_λ_^η^ B is LARGE AND ∇_λ_^η^ R2 is LARGE))
	AND ((∇_λ_^η^B is LARGE AND ∇_λ_^η^R3 is LARGE) OR (∇_λ_^η^B is LARGE AND ∇_λ_^η^R4 is LARGE))
	AND ((∇_λ_^η^B is LARGE AND ∇_λ_^η^R5 is LARGE) OR (∇_λ_^η^B is LARGE AND ∇_λ_^η^R6 is LARGE)),
	**THEN** the fuzzy gradient value ∇_λ_^ηF^ is LARGE.

**Fuzzy Rule 2**. Defining the fuzzy noisy factor Γ_*η*_	**IF MAX** ((∇_N_^η^) is LARGE, **M** **A** **X**((∇_S_^η^) is LARGE, **M** **A** **X**((∇_E_^η^) is LARGE, **M** **A** **X**((∇_W_^η^) is LARGE,
	**MAX** ((∇_SW_^η^) is LARGE, **M** **A** **X**((∇_NE_^η^) is LARGE,
	**MAX** ((∇_NW_^η^) is LARGE, and **M** **A** **X** ((∇_SE_^η^) is LARGE)))))))),
	**THEN** the noisy factor Γ_*η*_ is LARGE.

**Fuzzy Rule 3**. Defining the membership degrees *NE*_*E*_*C*_^R^_ for the red component *E*_*C*_^R^ in the fuzzy set “noise free"	**IF** (τ^R^ is LARGE AND τ^RG^ is LARGE AND τ^G^ is LARGE)
	OR (τ^R^ is LARGE AND τ^RB^ is LARGE AND τ^B^ is LARGE),
	**THEN** the noise-free degree of *E*_C_^R^ is LARGE, where the conjunction
	(A AND B) = A · B and the disjunction (A) OR (*B*) = A + B − A · B

**Fuzzy Rule 4**. Defining the weight *WE*_*C*_^R^ for the red component *E*_*C*_^R^	**IF** (*NE*_*E*_C_^R^_ is LARGE), **THEN ** *WE*_C_^R^ is LARGE.

**Fuzzy Rule 5**. Defining the weight *WE*_*λ*_^R^ for the neighbor of the red component *E*_*λ*_^R^	**I** **F** (*NE*_*E*_C_^R^_ is NO LARGE AND *NE*_*E*_λ_^R^_ is LARGE AND τ(Δ*E*_λ_^G^) is LARGE AND *NE*_*E*_λ_^G^_ is LARGE)
	OR (*NE*_*E*_C_^R^_ is NO LARGE AND *NE*_*E*_λ_^R^_ is LARGE AND τ(Δ*E*_λ_^B^) is LARGE AND *NE*_*E*_λ_^B^_ is LARGE),
	**THEN** *W*_*E*_λ_^R^_ is LARGE.

**Fuzzy Rule 6**. Defining the vectorial fuzzy gradient value ∇*δE*_*λ*_^*ηF*^ into the fuzzy similarity set LARGE	**I** **F** (∇δ*E*_λ_^η^B is LARGE AND ∇δ*E*_λ_^η^R1 is LARGE) OR (∇δ*E*_λ_^η^B is LARGE AND ∇δ*E*_λ_^η^R2 is LARGE)
	AND (∇δ*E*_λ_^η^B is LARGE AND ∇δ*E*_λ_^η^R3 is LARGE) OR (∇δ*E*_λ_^η^B is LARGE AND ∇δ*E*_λ_^η^R4 is LARGE)
	AND (∇δ*E*_λ_^η^B is LARGE AND ∇δ*E*_λ_^η^R5 is LARGE) OR (∇δ*E*_λ_^η^B is LARGE AND ∇δ*E*_λ_^η^R6 is LARGE),
	**THEN** the fuzzy similarity value ∇δ*E*_λ_^ηF^ is LARGE.

**Fuzzy Rule 7**. Defining the fuzzy noisy factor *ε*_*η*_	**IF** (**M** **A** **X**((∇δ_N_^η^) is LARGE, **M** **A** **X**((∇δ_S_^η^) is LARGE, **M** **A** **X**((∇δ_E_^η^) is LARGE, **M** **A** **X**((∇δ_W_^η^) is LARGE,
	**MAX** ((∇δ_SW_^η^) is LARGE, **M** **A** **X** ((∇δ_NE_^η^) is LARGE, and **MAX** (∇δ_NW_^η^ is LARGE, ∇δ_SE_^η^ is LARGE)))))))
	**THEN** the noisy factor ε_η_ is LARGE.

**Fuzzy Rule 8**. Defining the weights for the *Alfa-TM * filter *WE*_*λ*_ in the case of motion for the central pixel located in (*t*) frame	**IF** (*NE*_*E*_c_^R^_ is LARGE), **THEN ** *WE*_*λ*_ is LARGE.

**Fuzzy Rule 9**. Defining the value of ∇_λ_^ηF^ for edges detection into the edge detection similarity fuzzy set LARGE	**IF** (∇_λ_^η^B is NO LARGE AND ∇_λ_^η^R1 is NO LARGE) OR (∇_λ_^η^ B is NO LARGE AND ∇_λ_^η^ R2 is NO LARGE)
	AND (∇_λ_^η^B is LARGE AND ∇_λ_^η^R3 is LARGE) OR (∇_λ_^η^B is LARGE AND ∇_λ_^η^R4 is LARGE)
	AND (∇_λ_^η^B is LARGE AND ∇_λ_^η^R5 is LARGE) OR (∇_λ_^η^B is LARGE AND ∇_λ_^η^R6 is LARGE),
	**THEN** the fuzzy edge detection similarity gradient value ∇_λ_^ηF^ is LARGE.

**Fuzzy Rule 10**. Defining the weights for the *Alfa-TM * filter *WE*_*λ*_^R^ in the case of edge detection for the red component *E*_*λ*_^R^ in the (*t*_th_) frame	**IF** (*NE*_*E*_C_^R^_ is LARGE), **THEN ** *WE*_*C*_^R^ is LARGE.

**Fuzzy Rule 11**. Defining the weights for the mean filter *WE*_*C*_^R^ in the case of plain areas detection for the red component *E*_*C*_^R^ in the (*t*_th_) frame	**IF** (*NE*_*E*_C_^R^_ is NO LARGE), **THEN ** *WE*_*C*_^R^ is NO LARGE.

**Table 2:** PSNR values (dB) for threshold parameters Th_1_/Th_2_.

Foreman 0.005				Tennis 0.005				Foreman 0.010				Tennis 0.010			
Th_1_/Th_2_	0.37	0.45	0.53	Th_1_/Th_2_	0.37	0.45	0.53	Th_1_/Th_2_	0.37	0.45	0.53	Th_1_/Th_2_	0.37	0.45	0.53
0.15	38.05	38.11	38.09	0.15	28.26	28.33	28.29	0.15	35.94	36.02	35.98	0.15	27.82	27.88	27.87
0.22	38.09	**38.16**	38.12	0.22	28.31	**28.37**	28.33	0.22	36.01	**36.07**	36.03	0.22	27.86	**27.94**	27.91
0.29	38.05	38.11	38.09	0.29	28.26	28.33	28.29	0.29	35.94	36.02	35.98	0.29	27.82	27.88	27.87

PSNR values (dB) for threshold parameters Th_1_/Th_2_. Bold values indicate the best results respectively.

**Table 3:** MAE values (dB) for threshold parameters Th_3_/Th_4_.

Flowers 0.003				Stefan 0.003				Flowers 0.010				Stefan 0.010			
Th_3_/Th_4_	0.16	0.21	0.26	Th_3_/Th_4_	0.16	0.21	0.26	Th_3_/Th_4_	0.16	0.21	0.26	Th_3_/Th_4_	0.16	0.21	0.26
0.32	7.54	7.49	7.51	0.32	6.11	5.99	6.08	0.32	9.81	9.74	9.78	0.32	8.79	8.72	8.74
0.37	7.47	**7.41**	7.46	0.37	6.04	**5.92**	5.99	0.37	9.73	**9.65**	9.70	0.37	8.73	**8.65**	8.69
0.42	7.54	7.49	7.51	0.42	6.11	5.99	6.08	0.42	9.81	9.74	9.78	0.42	8.79	8.72	8.74

PSNR values (dB) for threshold parameters Th_1_/Th_2_. Bold values indicate the best results respectively.

**Table 4:** SSIM values (dB) for ∇_1_ and ∇_2_ parameters in membership functions.

Tennis 0.003				Stefan 0.005				Tennis 0.005				Stefan 0.015			
∇_1_/∇_2_	8	12	16	∇_1_/∇_2_	8	12	16	∇_1_/∇_2_	8	12	16	∇_1_/∇_2_	8	12	16
65	0.9346	0.9351	0.9348	65	0.9004	0.9013	0.9008	65	0.8652	0.8659	0.8655	65	0.8037	0.8046	0.8041
75	0.9350	**0.9356**	0.9352	75	0.9009	**0.9019**	0.9014	75	0.8657	**0.8664**	0.8660	75	0.8042	**0.8053**	0.8047
85	0.9346	0.9351	0.9348	85	0.9004	0.9013	0.9008	85	0.8652	0.8659	0.8655	85	0.8037	0.8046	0.8041

PSNR values (dB) for threshold parameters Th_1_/Th_2_. Bold values indicate the best results respectively.

**Table 5:** PSNR values (dB) for ∇_2,*η*_1_*η*_2__ and *δ*_*η*_1_*η*_2__^2^ parameters in membership functions*. *

Foreman 0.000				Flowers 0.010				Tennis 0.015				Stefan 0.020			
∇_2,*η*_1_*η*_2__/*δ*_*η*_1_*η*_2__^2^	0.002	0.004	0.53	∇_2,*η*_1_*η*_2__/*δ*_*η*_1_*η*_2__^2^	0.002	0.004	0.005	∇_2,*η*_1_*η*_2__/*δ*_*η*_1_*η*_2__^2^	0.002	0.004	0.005	∇_2,*η*_1_*η*_2__/*δ*_*η*_1_*η*_2__^2^	0.002	0.004	0.005
0.02	39.70	39.75	39.72	0. 02	26.73	26.77	26.78	0. 02	27.22	27.27	27.26	0.02	29.94	30.02	29.99
0.03	39.74	**39.78**	39.76	0.03	26.78	**26.83**	26.80	0.03	27.26	**27.31**	27.29	0.03	29.98	**30.07**	30.03
0.04	39.70	39.75	39.72	0.04	26.73	26.77	26.78	0.04	27.22	27.27	27.26	0.04	29.94	30.02	29.99

PSNR values (dB) for threshold parameters Th_1_/Th_2_. Bold values indicate the best results respectively.

**Table 6:** Average per 50 frames values for PSNR, MAE, NCD, and SSIM criteria obtained on the color video Flowers processed by RFMDAF [36], FDARTF_G [46], WMVCE [27], 3D-LLMMSE [26], NLM [52], VBM3D [50], and the proposed FMANS 3D filter. Bold values indicate the best results, respectively, for each noise level.

Flowers video sequence														
	RFMDAF		FDARTF_G		WMVCE		3D-LLMMSE		NLM		BM3D		FMANS	
*σ* ^2^	PSNR (dB)	MAE	PSNR (dB)	MAE	PSNR (dB)	MAE	PSNR (dB)	MAE	PSNR (dB)	MAE	PSNR (dB)	MAE	PSNR (dB)	MAE
0.000	28.51	7.10	28.13	7.31	28.69	6.93	28.14	7.45	28.83	6.65	28.92	6.54	**29.38**	**6.39**
0.003	27.28	8.41	27.56	8.25	27.50	8.19	27.07	8.32	28.46	7.63	28.54	7.56	**28.98**	**7.41**
0.005	26.59	9.30	26.67	8.80	27.22	8.43	26.22	8.99	27.69	7.96	27.81	7.78	**28.17**	**7.54**
0.010	25.08	11.95	25.40	10.65	25.24	10.31	24.77	10.81	26.37	9.94	26.44	9.81	**26.83**	**9.65**
0.015	23.85	13.84	24.24	12.30	24.29	11.58	23.83	12.62	24.99	10.63	25.03	10.52	**25.46**	**10.37**
0.020	22.98	14.30	23.42	13.56	23.82	12.65	23.51	13.87	24.16	11.73	24.21	11.58	**24.58**	**11.33**
0.030	21.70	16.46	22.38	15.27	22.43	14.23	21.91	15.64	22.93	13.29	23.04	13.15	**23.37**	**12.96**

*σ* ^2^	NCD	SSIM	NCD	SSIM	NCD	SSIM	NCD	SSIM	NCD	SSIM	NCD	SSIM	NCD	SSIM

0.000	0.014	0.8532	0.014	0.8532	0.014	0.8621	0.016	0.8521	0.011	0.8861	0.011	0.8869	**0.011**	**0.8882**
0.003	0.015	0.8181	0.015	0.8181	0.015	0.8217	0.017	0.8212	0.012	0.8432	0.012	0.8441	**0.012**	**0.8451**
0.005	0.016	0.7975	0.016	0.7975	0.016	0.7996	0.018	0.7985	0.016	0.8179	0.015	0.8190	**0.015**	**0.8192**
0.010	0.023	0.7248	0.023	0.7248	0.020	0.7354	0.023	0.7304	0.019	0.7509	0.019	0.7515	**0.019**	**0.7523**
0.015	0.024	0.6873	0.024	0.6873	0.023	0.6936	0.025	0.6914	0.020	0.7136	0.019	0.7143	**0.019**	**0.7148**
0.020	0.028	0.6395	0.028	0.6395	0.026	0.6489	0.027	0.6422	0.021	0.6659	0.020	0.6665	**0.020**	**0.6672**
0.030	0.031	0.6033	0.031	0.6033	0.028	0.6094	0.029	0.6035	0.023	0.6312	0.022	0.6319	**0.022**	**0.6323**

**Table 7:** Average per 50 frames values for PSNR, MAE, NCD, and SSIM criteria obtained on the color video Stefan processed by RFMDAF, FDARTF_G, WMVCE, 3D-LLMMSE, NLM, VBM3D, and the proposed FMANS 3D filter. Bold values indicate the best results, respectively, for each noise level.

Stefan video sequence														
	RFMDAF		FDARTF_G		WMVCE		3D-LLMMSE		NLM		BM3D		FMANS	
*σ* ^2^	PSNR (dB)	MAE	PSNR (dB)	MAE	PSNR (dB)	MAE	PSNR (dB)	MAE	PSNR (dB)	MAE	PSNR (dB)	MAE	PSNR (dB)	MAE
0.000	35.83	5.18	35.68	5.36	36.01	5.06	35.85	5.14	36.39	4.82	36.46	4.74	**36.83**	**4.62**
0.003	34.70	6.65	34.86	6.59	35.09	6.52	34.84	6.71	35.52	6.23	35.62	6.11	**35.94**	**5.92**
0.005	32.90	7.84	33.25	7.70	33.87	7.62	33.68	7.75	34.38	7.21	34.41	7.07	**34.80**	**6.89**
0.010	31.16	10.95	31.69	9.70	32.08	9.35	31.86	9.47	32.41	8.88	32.53	8.79	**32.95**	**8.65**
0.015	30.80	11.38	31.49	10.47	31.84	10.21	31.52	10.35	32.22	9.75	32.33	9.68	**32.69**	**9.51**
0.020	28.28	11.95	28.84	11.31	29.25	11.15	28.72	11.26	29.57	10.69	29.65	10.62	**30.07**	**10.48**
0.030	25.61	13.36	26.26	12.82	26.82	12.62	26.18	12.81	27.32	12.14	27.39	12.03	**27.83**	**11.83**

*σ* ^2^	NCD	SSIM	NCD	SSIM	NCD	SSIM	NCD	SSIM	NCD	SSIM	NCD	SSIM	NCD	SSIM

0.000	0.020	0.9463	0.020	0.9454	0.019	0.9538	0.020	0.9512	0.018	0.9586	0.018	0.9595	**0.018**	**0.9611**
0.003	0.023	0.9212	0.021	0.9228	0.021	0.9312	0.022	0.9287	0.019	0.9345	0.019	0.9356	**0.019**	**0.9372**
0.005	0.23	0.8850	0.022	0.8876	0.022	0.8921	0.023	0.8895	0.021	0.8996	0.020	0.9005	**0.019**	**0.9019**
0.010	0.026	0.8255	0.025	0.8279	0.024	0.8317	0.025	0.8278	0.023	0.8472	0.022	0.8486	**0.022**	**0.8498**
0.015	0.029	0.7802	0.027	0.7830	0.026	0.7954	0.027	0.7886	0.026	0.8026	0.025	0.8038	**0.024**	**0.8053**
0.020	0.032	0.7406	0.030	0.7430	0.029	0.7559	0.031	0.7457	0.028	0.7706	0.027	0.7721	**0.026**	**0.7737**
0.030	0.034	0.7030	0.033	0.7074	0.032	0.7186	0.033	0.7082	0.031	0.7241	0.029	0.7256	**0.028**	**0.7271**

**Table 8:** Average per 50 frames values for PSNR, MAE, NCD, and SSIM criteria obtained on the color video Foreman processed by RFMDAF, FDARTF_G, WMVCE, 3D-LLMMSE, NLM, VBM3D, and the proposed FMANS 3D filter. Bold values indicate the best results, respectively, for each noise level.

Foreman video sequence														
	RFMDAF		FDARTF_G		WMVCE		3D-LLMMSE		NLM		BM3D		FMANS	
*σ* ^2^	PSNR (dB)	MAE	PSNR (dB)	MAE	PSNR (dB)	MAE	PSNR (dB)	MAE	PSNR (dB)	MAE	PSNR (dB)	MAE	PSNR (dB)	MAE
0.000	38.68	4.67	38.41	4.82	38.89	4.48	38.61	4.52	39.43	3.91	39.50	3.78	**39.78**	**3.72**
0.003	37.75	5.48	37.88	5.32	38.42	5.09	38.34	5.18	39.11	4.56	39.22	4.47	**37.47**	**4.46**
0.005	36.61	6.60	36.91	6.25	37.11	5.79	37.02	5.92	37.71	5.43	37.92	5.36	**38.16**	**5.32**
0.010	34.40	9.00	34.86	8.59	35.09	7.51	34.82	7.67	35.63	7.06	35.80	6.98	**36.07**	**6.91**
0.015	33.18	10.20	33.69	9.79	33.95	9.14	33.79	9.45	34.51	8.65	34.64	8.51	**34.93**	**8.47**
0.020	32.11	11.84	32.55	11.26	32.74	10.39	32.56	10.74	33.29	9.83	33.38	9.74	**33.71**	**9.72**
0.030	29.25	13.15	29.90	12.73	30.19	11.88	30.05	12.07	30.78	11.13	30.89	11.04	**31.22**	**11.01**

*σ* ^2^	NCD	SSIM	NCD	SSIM	NCD	SSIM	NCD	SSIM	NCD	SSIM	NCD	SSIM	NCD	SSIM

0.000	0.010	0.9309	0.012	0.9289	0.010	0.9385	0.011	0.9321	0.009	0.9537	0.009	0.9543	**0.009**	**0.9552**
0.003	0.013	0.9058	0.013	0.9082	0.013	0.9122	0.013	0.9087	0.010	0.9199	0.010	0.9317	**0.010**	**0.9207**
0.005	0.015	0.8784	0.014	0.8811	0.014	0.8924	0.013	0.8818	0.011	0.9024	0.011	0.9042	**0.011**	**0.9032**
0.010	0.018	0.8301	0.017	0.8316	0.016	0.8419	0.027	0.8314	0.013	0.8502	0.013	0.8521	**0.013**	**0.8514**
0.015	0.021	0.7906	0.019	0.7941	0.018	0.8052	0.028	0.7936	0.016	0.8138	0.016	0.8153	**0.015**	**0.8149**
0.020	0.023	0.7598	0.021	0.7634	0.020	0.7775	0.021	0.7628	0.017	0.7861	0.016	0.7879	**0.016**	**0.7877**
0.030	0.025	0.7236	0.023	0.7267	0.022	0.7362	0.023	0.7261	0.020	0.7427	0.019	0.7435	**0.019**	**0.7438**

**Table 9:** Average per 50 frames values for PSNR, MAE, NCD, and SSIM criteria obtained on the color video Tennis processed by RFMDAF, FDARTF_G, WMVCE, 3D-LLMMSE, NLM, VBM3D, and the proposed FMANS 3D filter. Bold values indicate the best results, respectively, for each noise level.

Tennis video sequence														
	RFMDAF		FDARTF_G		WMVCE		3D-LLMMSE		NLM		BM3D		FMANS	
*σ* ^2^	PSNR (dB)	MAE	PSNR (dB)	MAE	PSNR (dB)	MAE	PSNR (dB)	MAE	PSNR (dB)	MAE	PSNR (dB)	MAE	PSNR (dB)	MAE
0.000	29.57	5.77	29.46	5.91	29.74	5.58	29.61	5.61	29.87	5.11	30.24	5.03	**30.56**	**4.87**
0.003	28.80	5.58	28.97	5.42	29.23	5.19	28.94	5.27	29.51	4.83	29.83	4.68	**30.13**	**4.56**
0.005	26.44	7.41	26.82	6.27	27.43	5.82	27.16	5.97	27.92	5.67	28.16	5.51	**28.46**	**5.35**
0.010	26.12	9.20	26.68	8.82	27.06	7.72	26.73	7.89	27.23	7.36	27.62	7.24	**27.87**	**7.12**
0.015	25.42	10.14	26.13	9.73	26.45	9.06	26.20	9.38	26.89	8.78	27.05	8.64	**27.30**	**8.41**
0.020	25.04	11.95	25.61	11.38	26.01	10.52	25.57	10.85	26.21	10.15	26.57	10.07	**26.83**	**9.89**
0.030	24.07	13.20	24.65	12.77	25.24	11.94	24.63	12.03	25.63	11.34	25.98	11.21	**26.23**	**11.04**

*σ* ^2^	NCD	SSIM	NCD	SSIM	NCD	SSIM	NCD	SSIM	NCD	SSIM	NCD	SSIM	NCD	SSIM

0.000	0.012	0.9368	0.011	0.9298	0.011	0.9384	0.011	0.9322	0.010	0.9437	0.010	0.9448	**0.010**	**0.9462**
0.003	0.013	0.9267	0.011	0.9228	0.012	0.9264	0.012	0.9246	0.012	0.9318	0.011	0.9331	**0.011**	**0.9356**
0.005	0.014	0.8427	0.014	0.8459	0.014	0.8523	0.014	0.8482	0.012	0.8627	0.013	0.8643	**0.012**	**0.8664**
0.010	0.017	0.8146	0.016	0.8184	0.015	0.8195	0.016	0.8174	0.013	0.8275	0.013	0.8289	**0.013**	**0.8315**
0.015	0.023	0.7764	0.020	0.7813	0.019	0.7857	0.020	0.7810	0.018	0.7917	0.018	0.7935	**0.017**	**0.7951**
0.020	0.026	0.7315	0.020	0.7412	0.020	0.7421	0.021	0.7417	0.018	0.7545	0.018	0.7554	**0.018**	**0.7579**
0.030	0.028	0.6936	0.022	0.7032	0.024	0.7069	0.023	0.6997	0.019	0.7193	0.021	0.7207	**0.020**	**0.7234**
